# Patterns of Lymph Node Pathology; Fine Needle Aspiration Biopsy as an Evaluation Tool for Lymphadenopathy: A Retrospective Descriptive Study Conducted at the Largest Hospital in Africa

**DOI:** 10.1371/journal.pone.0130148

**Published:** 2015-06-19

**Authors:** Denasha Lavanya Reddy, Willem Daniel Francois Venter, Sugeshnee Pather

**Affiliations:** 1 Department of Internal Medicine, Chris Hani Baragwanath Academic Hospital, University of the Witwatersrand, Johannesburg, South Africa; 2 Wits Reproductive Health and HIV Institute (RHI) Associate Professor, Department of Medicine, Faculty of Health Sciences, University of the Witwatersrand, Johannesburg, South Africa; 3 Division of Anatomical Pathology, School of Pathology, National Health Laboratory Service, Chris Hani Baragwanath Academic Hospital, Faculty of Health Sciences, University of the Witwatersrand, Johannesburg, South Africa; University of Cape Town, SOUTH AFRICA

## Abstract

**Background:**

Lymphadenopathy is a common clinical presentation of disease in South Africa (SA), particularly in the era of Human Immunodeficiency Virus (HIV) and tuberculosis (TB) co-infection.

**Methods:**

Data from 560 lymph node biopsy reports of specimens from patients older than 12 years at Chris Hani Baragwanath Academic Hospital (CHBAH) between 1 January 2010 and 31 December 2012 was extracted from the National Health Laboratory Service (NHLS), division of Anatomical Pathology. Cytology reports of lymph node fine needle aspirates (FNAs) performed prior to lymph node biopsy in 203 patients were also extracted from the NHLS. Consent was not obtained from participants for their records to be used as patient information was anonymized and de-identified prior to analysis.

**Results:**

The majority of patients were female (55%) and of the African/black racial group (90%). The median age of patients was 40 years (range12–94). The most common indication for biopsy was an uncertain diagnosis (more than two differential diagnoses entertained), followed by a suspicion for lymphoma, carcinoma and TB. Overall, malignancy constituted the largest biopsy pathology group (39%), with 36% of this group being carcinoma and 27% non-Hodgkin lymphoma. 22% of the total sampled nodes displayed necrotizing granulomatous inflammation (including histopathology and cytology demonstrating definite, and suspicious for mycobacterial infection), 8% comprised HIV reactive nodes; in the remainder no specific pathology was identified (nonspecific reactive lymphoid hyperplasia). Kaposi sarcoma (KS) accounted for 2.5% of lymph node pathology in this sample. Concomitant lymph node pathology was diagnosed in four cases of nodal KS (29% of the subset). The co-existing pathologies were TB and Castleman disease. HIV positive patients constituted 49% of this study sample and the majority (64%) of this subset had CD4 counts less than 350 cells/ul. 27% were HIV negative and in the remaining nodes, the HIV status of patients was unknown. The most common lymph node pathologies in HIV positive patients were Mycobacterial infection (31%), HIV reactive nodes (15%), non-Hodgkin lymphoma (15%) and nonspecific reactive lymphoid hyperplasia (15%). Only 8.7% were of Hodgkin lymphoma. In contrast, the most common lymph node pathologies in HIV negative patients were nonspecific reactive lymphoid hyperplasia (45%), carcinoma (25%) and Mycobacterial infection (11%). In this group, non-Hodgkin lymphoma and Hodgkin lymphoma constituted 9% and 8%, respectively. There were more cases of high-grade non-Hodgkin lymphoma in the HIV positive group compared to the HIV negative group. FNA and lymph node biopsy had statistically significant good agreement with regard to Hodgkin lymphoma (K 0.774, SE 0.07, 95% CI 0.606-0.882, p=0.001), non-Hodgkin lymphoma (K 0.640, SE 0.07, 95% CI 0.472-0.807, p=0.001), carcinoma (K 0.723, SE 0.069, 95% CI 0.528-0.918, p=0.001), and mycobacterial infection (K 0.726, SE 0.07, 95% CI 0.618-0.833, p=0.001).

**Conclusions:**

The most common lymph node pathologies in CHBAH are malignancies, nonspecific reactive lymphoid hyperplasia, necrotizing granulomatous inflammation and HIV reactive nodes. The distribution of disease differs in HIV positive patients. Overall, adequate FNA samples of lymph nodes have been found to have good correlation with lymph node biopsy findings in our setting.

## Introduction

Lymphadenopathy is a common presentation of disease in South Africa (SA), particularly in the era of Human Immunodeficiency Virus (HIV) and tuberculosis (TB) co-infection. There are only a limited number of studies that have described lymph node pathology in Southern Africa. Chris Hani Baragwanath Academic Hospital (CHBAH) which is a provincial tertiary hospital in Soweto, Johannesburg, contains close to 3300 beds and is the largest hospital in Africa [1].

In a post-mortem study performed in Johannesburg, TB was found to be the cause of death in the majority of patients (69%) with advanced Acquired Immunodeficiency Syndrome (AIDS), both before and after starting antiretroviral therapy [2]. Lymph nodes were the fourth most common site of positive mycobacterial cultures in this study (16%), following liver, spleen and lung [2]. The study also suggested that TB often goes unrecognized in these patients, and can be accompanied by other infections or neoplasms [2].

Excisional biopsy of a clinically appropriate lymph node is traditionally favored as a diagnostic procedure for a multitude of infectious and neoplastic disorders. However, fine-needle aspiration (FNA) is replacing it as a first line diagnostic procedure, reserving excisional biopsy for non-diagnostic FNA results that require further investigation [3–4].

We conducted a retrospective review of the lymph node pathology identified at CHBAH to contribute to medical knowledge of common causes of lymphadenopathy requiring biopsy in South Africa, and the role of FNA as an adjunct to excisional lymph node biopsy.

## Methods

The study was a retrospective descriptive audit conducted at Chris Hani Baragwanath Academic Hospital, Johannesburg, South Africa. The study population consisted of all patients over the age of 12 years who underwent lymph node biopsies at CHBAH between 1 January 2010 and 31 December 2012. Lymph node biopsy reports were extracted from the database of the National Health Laboratory Service (NHLS) Division of Anatomical Pathology.

Patient demographic details were obtained from the laboratory requisition forms submitted by attending clinicians and from pathology reports. There was no review of patient charts or files. Further information regarding HIV status (determined with antibody testing using 4^th^ generation HIV enzyme-linked immunosorbent assay), CD4 count (cells/ul), if relevant, and FNAs performed (smears in our setting) were accessed through the NHLS DisaLab system, using the patients’ name and hospital number. Consent was not obtained from participants for their records to be used as patient information was anonymized and de-identified prior to analysis.

All lymph node biopsy and FNA biopsy specimens were analyzed by pathologists employed by the NHLS at CHBAH, including one of the authors. Unfortunately, the authors reviewed not all the slides during this retrospective study. Histologic diagnoses were based on morphologic findings and, in the appropriate context, ancillary tests including Mycobacterial staining and culture, immunohistochemistry, FISH (fluorescent in-situ hybridization) techniques, and flow cytometry. The World Health Organization diagnostic criteria were applied to the histopathology demonstrated on lymph node biopsies. The diagnosis of tuberculous lymphadenitis was confirmed by Ziehl-Neelsen stain for acid fast bacilli and positive Mycobacterial culture. Mycobacterial culture and Mycobacterial PCR testing using Xpert MTB/RIF are performed in cases of clinically and histopathologically (granulomatous) suspected Mycobacterial lymphadenitis. Ziehl-Neelsen stains for acid fast bacilli were routinely performed on all lymph node biopsies and FNA biopsies that displayed granulomatous inflammation (necrotizing and non-necrotizing). HIV lymphadenitis was graded as follows: grade 1 if associated with hyperplastic features and enlargement of germinal centres with increased apoptosis and phagocytosis by macrophages; grade 2 if there was a reduction in lymphoid follicles and mature lymphocytes, but an increase in plasma cells and perifollicular blood vessels; and grade 3 if the germinal centres were sclerotic. HIV lymphadenitis was not used as a surrogate marker for HIV infection, as it describes a histological pattern not entirely specific to HIV.

There were no specific exclusion criteria. Specimens deemed unsuitable for histopathologic or cytologic analysis by the pathologist were excluded. Unsuitable samples were attributed to tissue samples not representative of lymphoid tissue or poor quality specimens that could not be evaluated. Non-diagnostic specimens were included in the analysis. In cases where a single patient had more than one lymph node biopsy during the period of the study, the repeat biopsies were evaluated as a subset. A total of 203 reports of FNAs preceding lymph node biopsy were evaluated. Findings of the FNA were analyzed in conjunction with adequacy of FNA samples submitted for cytology, prior to comparison with biopsy. Of the FNAs, 23% were inadequate for assessment, 76% were adequate and in 1% adequacy was not commented on.

Statistical analysis was performed using STATA version 12 for Windows (StataCorp LP, Texas; www.stata.com) and GraphPadQuickCalcs (GraphPad Software Inc, California; www.graphpad.com). A statistician was consulted. The Human Research Ethics committee at the University of the Witwatersrand granted ethical approval of this study (Clearance Certificate number M130626).

## Results

The majority of patients (55%) who had lymph node biopsies were female (Table 1). Over 90% of patients were of the African/black racial group, in keeping with the racial demographic distribution in SA. The median age of patients was 40 years, with a minimum of 12 and a maximum of 94. The most common indication for biopsy was an uncertain diagnosis (more than two differential diagnoses entertained), followed by a suspicion for lymphoma, carcinoma and TB (Table 2).

**Table 1:** Incisional, Excisional and Core Biopsy Diagnosis in relation to Demographics.

Demographic	Malignancy, n = 219							Necrotizing nodes, n = 125	HIV^1^ React nodes, n = 46	Not definitive, n = 170	TOTAL, n = 560
		KS^2^	NHL^3^	CHL^4^	Car^5^	Other	Subtotal				n(%)
GENDER	Male	9	29	27	21	11	97	58	17	80	252(45)
	Female	5	31	22	58	6	122	67	29	90	308(55)
RACE	Asian				1		1	2			3(0.5)
	Black	14	58	46	69	16	203	116	44	160	523(93.3)
	White			2	9		11	3	1	9	24(4.2)
	Mixed Race		2	1		1	4	4	1	1	10(2)
AGE	Median 40										
	(12–94)										
AGE BAND	12-20yr		4	5	2		11	11	1	18	41(7.3)
	21-35yr	11	13	20	13	9	66	59	22	40	187(33.4)
	36-50yr	3	25	17	31	6	82	46	20	53	201(36)
	51-65yr		15	7	25	1	48	8	3	48	107(19)
	>65yr		3		8	1	12	1		11	24(4.3)

^1^HIV: Human Immunodeficiency Virus

^2^KS: Kaposi sarcoma

^3^NHL: non-Hodgkin Lymphoma

^4^CHL: Hodgkin Lymphoma

^5^Car: carcinoma.

**Table 2:** Incisional, Excisional and Core Biopsy Diagnosis in relation to Indications and site of biopsy.

		Malignancy, n = 219						Necrotizing nodes, n = 125	HIV^1^ React nodes, n = 46	Not definitive, n = 170	TOTAL, n = 560
		KS^2^	NHL^3^	CHL^4^	Car^5^	Other	Subtotal				n(%)
INDICATION FOR BIOPSY	Suspect TB^6^	1	5	2	2	1	11	46	9	8	74(13)
	Suspect lymphoma	2	33	28	4	4	71	17	13	19	120(22)
	Staging Car^5^				60		60			54	114(20)
	Uncertain Diagnosis	9	16	14	12	9	60	45	20	77	202(36)
	Unknown	2	6	5	1	3	17	17	4	12	50(9)
SITE OF LYMPH NODE BIOPSY	Cervical	8	26	37	18	9	98	73	16	31	218(40)
	Inguinal	2	10	2	10	1	25	5	9	19	58(10)
	Axilla	2	11	7	35	6	61	18	12	38	129(23)
	Other	2	13	3	16	1	35	29	9	82	155(27)

^1^HIV: Human Immunodeficiency Virus

^2^KS: Kaposi sarcoma

^3^NHL: non-Hodgkin Lymphoma

^4^CHL: Hodgkin Lymphoma

^5^Car: carcinoma

^6^TB: Tuberculosis.

The most frequent site of biopsy was cervical, followed by “other” which included laparoscopic biopsies (of intra-abdominal nodes), axillary and inguinal nodal biopsies. Laparoscopic biopsies were performed as part of a staging or curative process for patients known to have carcinoma. In other instances, lymph nodes were found incidentally at laparotomy or laparoscopy and sampled.

Interestingly, of the suspected lymphoma cases, 28% were diagnosed with non-Hodgkin lymphoma, and 23% with Hodgkin lymphoma. Of the suspected TB cases, 62% displayed necrotizing granulomatous inflammation on biopsy, while 53% of staging carcinoma had confirmed carcinoma on biopsy.

Overall, malignancy was the largest biopsy pathology group (39%), with 36% of this group having carcinoma and 27% non-Hodgkin lymphoma. 22% of the total sample comprised nodes that displayed necrotizing granulomatous inflammation (including histopathology demonstrating definite mycobacterial infection and suspicious for mycobacterial infection). 8% of the sample comprised HIV reactive nodes and in the remainder no specific pathology was identified (including nonspecific reactive lymphoid hyperplasia).

KS accounted for 2.5% of lymph node pathology in the sample. Concomitant lymph node pathology was diagnosed within four cases of nodal KS (29% of the subset). The co-existing pathologies were mycobacterial infection in one patient, Castleman disease in two patients, and a combination of mycobacterial infection and Castleman disease in the fourth patient.

HIV positive patients constituted 49% of the sample, with the majority of patients in this subset (64%) having a CD4 count below 350 cells/ul. 27% were HIV negative and in the remaining nodes, the HIV status of patients was unknown. The most common lymph node pathologies in HIV positive patients were mycobacterial infection (31%), HIV reactive nodes (15%), non-Hodgkin lymphoma (15%) and non-specific reactive lymphoid hyperplasia (15%) (Table 3). Only 8.7% were of Hodgkin lymphoma. In contrast, the most common lymph node pathologies in HIV negative patients were nonspecific reactive lymphoid hyperplasia (45%), carcinoma (25%) and Mycobacterial infection (11%). In this group, non-Hodgkin lymphoma and Hodgkin lymphoma constituted 9% and 8%, respectively.

**Table 3:** Incisional, Excisional and Core Biopsy diagnosis in relation to HIV status.

HIV Status		Malignancy, n = 219						Necrotizing nodes, n = 125	HIV^1^ React nodes, n = 46	Not definitive, n = 170	TOTAL, n = 560
		KS^2^	NHL^3^	CHL^4^	Car^5^	Other	Subtotal				n(%)
	Subtotals	14	60	49	79	17	219	125	46	170	560(100)
HIV^1^ NEGATIVE			14	12	37	1	64	17	1	68	150(27)
HIV^1^ UNKNOWN		1	6	13	28	1	49	23	4	60	136(24)
HIV^1^ POSITIVE		13	40	24	14	15	106	85	41	42	274(49)
CD4^6^ BAND (cells/ul)	<100		7	4		3	14	35	2	6	57(10)
	100–200	3	13	5	5	2	28	19	5	6	58(10.4)
	201–350	5	8	8	1	6	28	10	12	11	61(11)
	351–500	2	2	5	1		10	1	7	4	22(4)
	>500	1	3		3	4	11	2	5	6	24(4.3)
	Nil/Unknown	2	7	2	4		15	18	10	9	52(9.3)

^1^HIV: Human Immunodeficiency Virus

^2^KS: Kaposi sarcoma

^3^NHL: non-Hodgkin Lymphoma

^4^CHL: Hodgkin Lymphoma

^5^Car: carcinoma

^6^CD4: Cluster of Differentiation 4 T-cells.

Amongst the cases with non-Hodgkin lymphoma, 66% were HIV positive. Of the HIV positive subset, 50% were diagnosed with Diffuse Large B-Cell Lymphoma (DLBCL) and 17.5% with Burkitt lymphoma. Plasmablastic lymphoma, Intermediate Burkitt/ DLBCL and Anaplastic Large Cell Lymphoma each contributed a further 10% to the HIV positive group. In contrast, DLBCL was found in only 21% of the HIV negative group. Only one HIV negative case was classified as HIV lymphadenitis. This diagnosis was influenced by incorrectly specified clinical information regarding HIV status in the laboratory request form. As mentioned earlier, HIV lymphadenitis is a histological pattern not entirely specific to HIV and therefore, cannot be used as a surrogate marker for HIV infection.

There were 203 (36%) patients in our study sample who also underwent FNAs prior to biopsy. Of the FNAs, 23% were inadequate for assessment, 76% were adequate and in 1% adequacy was not commented on. When compared with the histopathology diagnosis as per lymph node biopsies, FNA was found to have statistically significant good agreement/reliability with regards to Hodgkin lymphoma (K 0.774, SE 0.07, 95% CI 0.606–0.882, p = 0.001), non-Hodgkin lymphoma (K 0.640, SE 0.07, 95% CI 0.472–0.807, p = 0.001), carcinoma (K 0.723, SE 0.069, 95% CI 0.528–0.918, p = 0.001), and mycobacterial infection (K 0.726, SE 0.07, 95% CI 0.618–0.833, p = 0.001) (Table 4).

**Table 4:** Statistical Agreement between LN FNA and LN Biopsy.

Pathological Diagnosis	% observed agreements	% agreements expected by chance	Kappa	SE	95% Confidence Interval	P-value
CHL^1^	94.09	76.92	0.744	0.07	0.606–0.882	0.001
NHL^2^	92.61	79.50	0.640	0.07	0.472–0.807	0.001
Car^3^	96.55	87.55	0.723	0.069	0.528–0.918	0.001
Granulom inflam NOS^4^	97.04	95.20	0.385	0.07	-0.005–0.775	0.001
MI^5^	89.16	60.49	0.726	0.07	0.618–0.833	0.001
Non-specific reactive LNH^6^	80.30	67.28	0.398	0.067	0.249–0.547	0.001

^1^CHL: Hodgkin Lymphoma

^2^NHL: non Hodgkin Lymphoma

^3^Car: carcinoma

^4^Granulom inflam NOS: Granulomatous Inflammation not otherwise specified

^5^MI: Mycobacterial Infection

^6^Non-specific reactive LNH: non-specific reactive lymph node hyperplasia.

There were two cases where a single patient had more than one lymph node biopsy during the period of the study. Both patients were HIV positive with CD4 counts below 200 cells/ul. FNAs were performed prior to biopsy in both cases and were not definitive. The sites of the biopsies were, respectively, axillary and cervical in the first patient, and cervical both times, in the second patient. The definitive biopsies were both repeated due to a high index of clinical suspicion, and subsequently confirmed to be non-Hodgkin lymphoma.

## Discussion

Our study affirmed local demographic patterns, in that the majority of patients who underwent lymph node biopsy at CHBAH were middle-aged African/black females. We have demonstrated that nodal pathology diagnoses at CHBAH are inclusive of malignancy, necrotizing granulomatous inflammation and reactive nodes (due to HIV or other). The most common lymph node pathologies at CHBAH as per biopsy are in keeping with local African literature [5–7]. However, our data is unique in that it also describes differences in pathology between HIV positive and negative patients, the former further stratified by CD4 count.

Our study highlights the known significant correlation between HIV incidence and incidence of aggressive B-cell lymphomas and Hodgkin lymphoma [8–9]. Associated risk factors for the development of these malignancies are high HIV viral loads and low CD4 counts, although malignancies may occur at any CD4 count. The pathogenesis is thought to be immune dysregulation with loss of T-cell immunity against onco-viruses like Epstein-Barr Virus and human herpesvirus 8 [8].

The most common indications for requesting a lymph node biopsy in our setting were an uncertain diagnosis and the suspicion of malignancy or TB, which reflects the relative dominance of the latter two diseases in Southern Africa. The global prevalence and death rates from TB are on the decline. However, in contrast, Africa is showing an increase in the tuberculous burden [10].

In a study performed at CHBAH, TB was diagnosed in 1291 patients over a period of two months [1]. The association between HIV infection and TB is well described in South Africa. The high prevalence of TB is also reflected by HIV statistics for SA [11]. South Africa carries the highest global burden of HIV with an estimated 5.6 million people infected, with TB being the most common serious opportunistic infection [11–13].

The previous CHBAH study showed that of the patients diagnosed with TB, 74% had pulmonary TB and of the patients with extrapulmonary TB, pleural and miliary were the most common forms [1]. However, more recent publications suggest that TB lymphadenitis is the most common form of extrapulmonary TB [3–4,14]. The high prevalence of TB and TB suspicious lymphadenitis at CHBAH has been described, notably in the age band 25–44 years [15]. In our study, 22% of lymph node biopsies and 27% of FNAs comprised nodes that displayed necrotizing granulomatous inflammation (including histopathology and cytology demonstrating definite mycobacterial infection and suspicious for mycobacterial infection), confirming the high prevalence of TB in CHBAH.

The study by Martinson et al stated that the risk of TB increases at lower CD4 counts, and suggested that early antiretroviral therapy reduced the population prevalence of TB in HIV infected patients [12]. This suggestion may also be evident in our study as the majority of HIV positive patients in our sample had CD4 counts less than 350 cells/ul and the commonest lymph node pathology in this group was confirmed and suspected TB.

There is a wide differential diagnosis for peripheral TB lymphadenitis. Patients with lymphoma in rural KwaZulu Natal were misdiagnosed based on clinical similarities to TB and were placed on empiric TB treatment, delaying the lymphoma diagnosis by a median of five months [16]. The dangers of misdiagnosing TB, and subsequent empiric TB treatment, are progression of underlying disease (malignancy or other infection); toxicity of TB therapy; and development of drug-resistant therapy [11, 17].

A Zambian study suggested that primary HIV lymphadenopathy was a significant cause of superficial lymphadenopathy [18]. This finding is also highlighted in our study, as HIV reactive nodes comprised 8% of the total sample, and was amongst the largest nodal pathology group in HIV positive patients (15%).

HIV-associated lymphadenitis (reported in our setting as HIV reactive nodes) is a well-characterized pattern of histological findings in lymph nodes of many HIV-infected individuals. It is likely due to the lymphotropism of the HI virus. Grade 1 is associated with hyperplastic features, enlargement of germinal centres with increased apoptosis and phagocytosis by macrophages. In Grade 2 there is a reduction in lymphoid follicles and mature lymphocytes, but an increase in plasma cells and perifollicular blood vessels. In Grade 3, the germinal centres become sclerotic [19].

Rare causes of lymphadenopathy in our study included one case of Cryptococcosis (microbiologically confirmed) in an HIV positive male patient who had profound immunosuppression (CD4 count of 8 cells/ul). In addition, there was a case of myeloid sarcoma (extramedullary counterpart of chronic myeloid leukaemia) in an HIV positive female who had a CD4 count of 1626 cells/ul.

The association between KS and multicentric Castleman disease due to the common causal agent human herpesvirus 8 (HHV8) is well described in the literature [20]. This association has been demonstrated by the co-existence of both these pathologies in lymph nodes in our study. Interestingly, two patients in our study with coexisting KS and Castleman disease had CD4 counts above 350 cells/ul. CD4 counts were not available for the remaining two patients who had concomitant lymph node pathology. A possible explanation for the relatively high CD4 counts may be immune reconstitution inflammatory syndrome.

We then evaluated the outcomes of fine needle aspiration biopsy in our setting. FNA is widely regarded as the diagnostic modality of choice in diagnosing TB lymphadenitis [10, 21]. Our study affirms a good correlation between FNA diagnosis and biopsy diagnosis on lymph node specimens. In the event of inadequate FNA samples, the result should be treated with reserve. If there is a clinical concern for nodal-based mycobacterial disease or malignancy despite a non-contributory FNA, a clinically appropriate lymph node should be submitted for histopathologic assessment.

In the event of a persistent clinical concern, despite a negative histopathology result, the nodal biopsy should be repeated with emphasis on selection of a clinically appropriate node. The importance of a repeat biopsy where there is a high clinical suspicion for disease, particularly in HIV positive patients, was highlighted in the two patients from our study subsequently diagnosed with non-Hodgkin lymphoma [16]. Our findings suggest that negative biopsy findings in HIV positive patients should be treated with reserve if there is a high index of clinical suspicion for lymphoma.

Limitations of the study include its selection bias, retrospective nature, and possible information bias as data was extracted from a database in which variable SNOMED codes for nodes at different topographic sites may be used. However, our findings correlate well with both local and international literature on the subject. In correlating FNA with excisional lymph node biopsy, there is scope for prospective studies to directly compare the two modalities and develop clinical algorithms taking into account the clinical and demographic profile of patients who should proceed directly to lymph node biopsy in order to expedite diagnosis and treatment.

## Conclusions

Our study showed that the most common lymph node pathologies occurring in patients who underwent biopsies at CHBAH are malignancies, nonspecific reactive lymphoid hyperplasia, necrotizing granulomatous inflammation due to mycobacterial infection, and HIV reactive nodes. The distribution of disease differs in HIV positive patients. FNA was found to have good overall correlation with histopathology biopsy diagnoses in our setting. The diagnosis specified in an FNA report should be interpreted in conjunction with the comment/s about adequacy of the aspirated specimen for assessment. Due to the coexistence of nodal KS with other pathologies, patients who have suspected nodal KS would benefit from proceeding directly to have biopsies for histopathological assessment.

## Supporting Information

S1 Dataset FNA Stratification. (XLS)
